# Aloe and its Effects on Cancer: A Narrative Literature Review

**DOI:** 10.24248/eahrj.v5i1.645

**Published:** 2021-06-11

**Authors:** Astère Manirakiza, Laurent Irakoze, Sebastien Manirakiza

**Affiliations:** a University Hospital Center of Kamenge, Burundi; b Chongqing Medical University; c Faculte de Medecine de Bujumbura, Universite du Burundi

## Abstract

Many years ago, *Aloe Vera* was cited to have a lot of therapeutic properties including; anti-microbial, anti-viral, anti-cancer, anti-oxidant, anti-inflammatory, skin protection, wound healing, and regulation of blood glucose and cholesterol. However, Aloe could present some side effects. This review focused on the latest discoveries regarding the therapeutic role of Aloe plant or its compounds on the acquired biological capabilities for tumour growth and progression namely; evading growth suppressor, avoiding immune destruction, enabling replicative immortality, tumour promoting inflammation, activating invasion and metastasis, inducing angiogenesis, genome instability and mutation, resisting cell death, deregulating cellular energetics and sustaining proliferating signalling. It clarified the anti-cancer activities it exerts on different types of cancer and also highlighted some pro-oncogenic pathways that can be disrupted by different compounds of Aloe.

## BACKGROUND

Around 420 species of Aloe are inventoried worldwide, but the most popular and widely used is *Aloe Barbadensis Miller* (also called *Aloe Vera* Linne, commonly referred to as *Aloe Vera*).^1,2^ For many years, Aloe is known to have many therapeutic properties which include; anti-microbial, anti-viral, anti-cancer, anti-oxidant, anti-inflammatory, skin protection, wound healing, and regulation of blood glucose and cholesterol.^3^

Several studies have illustrated the role of Aloe in cancer prevention and treatment, around 75 active compounds could potentially be of therapeutic value in cancer treatment.^4^ Even though Aloe or its compounds is known play anti-cancer activities in many cancer types in vitro, few studies have reported this evidence. Furthermore, many in vitro studies have demonstrated the effectiveness of the whole Aloe or its compounds in inhibiting the proliferation or growth of tumours.

Whole Aloe could have an inherent anti-tumour activity because of its many compounds and could be involved in the disrupting of tumour growth and progression signalling pathways. This mechanism of action could invariably inhibit the growth of cancer cells and lead to good prognosis. However, there are some controversies about toxicities of Aloe given the recent review which reported the side effects of Aloe especially for *Aloe Vera* on neoplastic and non-neoplastic cells.^1^

Many acquired capabilities are necessary for tumour growth and progression, namely; Evading growth suppressor, Avoiding immune destruction, enabling replicative immortality, tumour promoting inflammation, activating invasion and metastasis, inducing angiogenesis, genome instability and mutation, resisting cell death, deregulating cellular energetics and sustaining proliferating signalling.^5^ In its anti-cancer activities, the aloe could act on one or more of these capabilities for tumour growth and progression.

Therefore, this narrative literature review aimed to present the effectiveness of Aloe or its compounds on cancers taking into account the acquired capabilities of Cancer.

### Identification of Relevant Studies and Research Method

We systematically searched on PubMed and google scholar databases. The combination of key words were Aloe and cancers, Aloe and tumours, Aloe and tumours suppressors, Aloe and cancer cytotoxicity, Aloe and cancer apoptosis, Aloe and tumour growth, Aloe and tumour proliferation, Aloe and tumour inflammation, Aloe and tumour and immune, Aloe and cancer metastasis, Aloe and cancer angiogenesis, Aloe and DNA cancer cells, Aloe and normal cells.

Through identified studies, we systematically identified any compound of Aloe which has any anticancer activity. To perform deeply our research, every time the item Aloe was replaced by the identified compound in the above combination. Other studies were identified through references.

### The Biological Capabilities of Cancer and Aloe

#### Antigrowth activity of Aloe on cancer cells

*Aloe Vera* proved its anticancer effect when it was administrated to rats with pleural tumour from hepatoma cells.^6^ The dichloromethane (CH_2_Cl_2_) extract of cape aloe (concentrated and dried leaves of various species of Aloe, mainly Aloe *ferox*) caused growth inhibitory effect in Ehrlich ascites tumour cells, a decrease in DNA synthesis and an accumulation of cells in the G_1_ phase.^7^

#### Cytotoxicity of Aloe on cancer cells

The Aloin is a natural anthracycline and it is known that anthracycline class medication such as doxorubicin is used in treatment of various types of cancer namely breast carcinoma, osteosarcoma and cancer of soft tissues, Hodgkin lymphoma, non-Hodgkin lymphoma, Solid tumour of children, lung cancers, acute and chronic leukaemia, bladder cancer, ovarian cancer and gastric cancer.^8,9^ Aloin's cytotoxicity effect was found and more marked in breast cancer cells without ErbB-2 than those with ErbB-2.^8^ The Emodin, a natural anthraquinone found in Aloe^10,11^ and in other plants was involved in a cytotoxic activities in human myeloma.^12^

#### Apoptosis and antiproliferative activities of Aloe

When murine myeloma cells were treated by leaf extract of *Aloe arborescens,* the antiproliferative activity was very high while in the control group of cells, the reverse activity was observed.^13^ Moreover, the anti-proliferative effect of total extract from leaves of *Aloe arborescens* (8%) was very high than the one of Aloe-emodin (natural hydroxyanthraquinone present in the leaves of *Aloe Vera*) in glioblastoma cells.^14^

Aloe-emodin showed its efficacy to inhibit proliferation and to induce apoptosis in many types of cancerous cells by various mechanisms (Table 1). They include human colon carcinoma cells, human oral squamous cell carcinoma, human gastric carcinoma cells, human colorectal cancer cells, human cervical cancer cells, human lung squamous carcinoma, human malignant glioma cells, human tongue squamous cancer cells, prostate cancer cells, human colon cancer cells, human nasopharyngeal carcinoma cells, human bladder cancer cells, and hepatocellular carcinoma cells.^15–30^

**TABLE 1:** The Compounds of Aloe and Mode of Action on Cancers

Compounds	Country	Experiments	Effects	Type of cancer	Mode of action	References
**Whole Aloe**						
Aloe Vera	Italy	Vivo	Anticancer	Pleural tumour from hepatoma	Not described	6
Aloe Vera	Egypt	Vitro	Anticancer & apoptosis	hepatocellular carcinoma	Increase P53 and decrease Bcl-2 genes expressions	37
Aloe Vera	Poland	Vivo	Anti-angiogenesis, photocytotoxicity	Sarcoma	Not described	54
Aloe Vera	Japan	Vitro	Suppression cell proliferation	Neuroblastoma	Probably by suppressing CCND2 transcript levels	65
Aloe Vera	UAE	Vitro	Inhibition of cancer cell growth	Breast and cervical cancer	Apoptotic pathway	95
Aloe Vera	USA	Vivo	Inhibition of tumour	Ocular Surface Squamous Neoplasia	Not described	105
Aloe Vera extract	Korean	Vitro	Induction of apoptosis	Hepatocellular carcinoma	ATP depletion-related impairment of mitochondria, which is caspase-independent	109
Aloe arb-orescens Miller	Japan	Vivo	Anticancer & ant-proliferative	Duodenal tumour	Not described	110
Aloe arborescens	Italy	Vitro/Vivo	Tumour grow th inhibition	Glioblastoma	Not described	14
**Aloe-emodin**						
Aloe-Emodin	Taiwan	Vitro	Antiproliferarative	Colon carcinoma	Inhibition of casein kinase II activity, The release of apoptosis-inducing factor and cytochrome c, Caspase-3 activation	15
Aloe-emodin	China	Vitro	Antiproliferative, increase apoptosis	Oral squamous carcinoma	Activation of caspase-9 and caspase-3 proteins	16
Aloe-emodin	Taiwan	Vitro	Induction of-apoptosis	Gastric carcinoma	Release the apoptosis-inducing factor and cytochrome c from mitochondria, Activation of caspase-3	17
Aloe-Emodin	China	Vitro	Suppression of cell viability induction of apoptosis, endoplasmic reticulum stress	Colorectal	Activation of factor C/EBP homologous protein and caspase-12	18
Aloe-Emodin	Poland	Vitro	Induction of apoptosis	Cervical Cancer	Mitotic catastrophe, inhibition of cell division in the G2/M phase, reduction of viability	19
Aloe-emodin (Nano)	China	Vitro/Vivo	Antiprolifercell carcinoma ative induction of cell cycle arrest & apoptosis, antitumour growth	Lung squamous	Cleavage of Caspase-3, poly (ADP-ribose), polymerase (PARP), Caspase-8 and Caspase-9, Enhanced reactive oxygen species (ROS) production	20
Aloe-emodin	Italy	Vitro/Vivo	Tumour growth inhibition	Glioblastoma	Reduction pAKT phosphorylation, block of cell cycle in S and G2/M phase	14
Aloe-emodin	Malaysia	Vitro	Induction of apoptosis and cell cycle arrest in S phase	Malignant glioma	Promotion of the loss of mitochondrial membrane potential	21
Aloe-emodin	China	Vitro	Induction of cell death through S-phase arrest and apoptosis	Tongue squamous carcinoma	Promotion of p53, p21 and p27, Promotion of the release of apoptosis-inducing factor, endonuclease G, pro-caspase-9 and cytochrome c	22
Aloe-emodin	Korea	Vitro/Vivo	Suppression of cancer progression	Prostate cancer	Binding with mTORC2 and inhibit its kinase activity	23
Aloe-emodin	India	Vitro	Induction of cell cycle arrest in G2/M phase & apoptosis	Colon cancer	Activation of Caspase-6	24
Aloe-emodin	Taiwan	Vitro	Induction of cell cycle arrest in G2/M phase & apoptosis	Nasopharyngeal carcinoma	Caspase-8-mediated activation of the mitochondrial death pathway	25
Aloe-emodin	China	Vitro	Induction of cell cycle arrest in G2/M phase & apoptosis	Bladder Cancer	Activation of p53, p21, Fas/APO-1, Bax and caspase-3.	26
Aloe-emodin	China	Vitro	Induction of-growth inhibitory through cell cycle arrest in G2/M phase	Cervical cancer	Cell cycle arrest in G2/M phase	27
Aloe-emodin	Taiwan	Vitro	Induction of cell cycle arrest in G1 phase and apoptosis	Hepatoma	Induction of p53 and p21 expression	28
Aloe-emodin	USA	Vitro	Inhibits proliferation, and induces apoptosis	Glioma	Delaying S phase progression, reduction of poly (ADP-ribose) polymerase and protein kinase C, cleavage of caspase 7	29
Aloe-emodin	Taiwan	Vitro	Induction of apoptosis	Lung squamous cell carcinoma	Activation of caspase-3, caspase-8, and caspase-9	30
Aloe-emodin	Italy	Vitro/Vivo	Induction of apoptosis	Neuroectodermal Tumours	Not well described	31
Aloe-emodin	Israel	Vivo	Inhibition of cells proliferation	Merkel cell carcinoma	Not described	32
Aloe-emodin	Italy	Vitro	Cells antiproliferative and differentiation	Leukaemia	Not described	38
Aloe-emodin	Taiwan	Vitro	Induction of cell cycle arrest in G2/M phase & antiproliferative	Promyelocytic leukaemia	Not described	39
Aloe-emodin	Italy	Vitro	Anticancer	Multidrug resistant leukaemia cells	Not described	40
Aloe-emodin	Spain	Vivo	Anti-angiogen-esis	Not specific	Inhibits endothelial cell proliferation	53
Aloe-emodin	India	Vitro	Inhibition of cell migration/angiogenesis	Colon cancer	Down-regulating of Matrix Metalloproteinase (MMP-2/9), RhoB and VEGF by reducing DNA binding activity of NF-kB	55
Aloe-emodin	China	Vitro	Suppression of the metastasis	Breast cancer	Inhibition of the capabilities of invasion and migration of cells probably	63
Aloe-emodin	Italy	Vitro	Anticancer and anti-proliferation	Melanoma	Decreasing the secretion of matrix mettalloprote-inase-9	66
Aloe-emodin	China	Vitro	Arrest the cell cycle in G2/M phase	Gastric cancer	Inhibition of the expressions of protein kinase C and c-myc	67
Aloe-emodin	Taiwan	Vitro	Induction of cells death	Lung non-small cell carcinoma	Decreasing Cyclic adenosine monophosphate (cAMP)-dependent protein kinase, protein kinase C, Bcl-2, caspase-3 and p38	111
Aloe-emodin	Taiwan	Vitro	Induction of DNA damage and apoptosis	Lung carcinoma	Production of generation of reactive oxygen species and decrease in the mRNA of DNA repair enzymes	69
Aloe-emodin	China	Vitro/Vivo	Antineoplastic (cell proliferation was blocked in G1 phase)	Oral mucosa carcinoma	Reactive oxygen species (ROS) generated and up-regulation of Caspase-3	99
Aloe-emodin	China	Vitro	Inhibition of tumour	Gastric cancer	Not described	100
Aloe-emodin	Serbia & Montenegro	Vivo	Anticancer, induction of apoptosis	Glioma	Inhibition of extra cellular signal-regulated kinases 1 and 2 (ERK1/2) independent induction	112
Aloe-emodin	Singapore	Vitro	Induction of apoptosis and cell cycle arrest in G2/M	Hepatocellular carcinoma	Induction of higher caspase-3-like activity	68
Aloe-emodin	China	Vitro	Anticancer	Tongue Cancer	Induction of DNA damage and inhibition of DNA repair gene expression	71
Aloe-emodin	Taiwan	Vitro	Suppression of breast cancer cell proliferation	Breast Cancer	Targeting estrogen receptor protein stability through distinct mechanisms	113
**Emodin**						
Emodin	Taiwan	Vivo	Cytotoxicity	Lung squamous cell carcinoma	Activation of caspase-3, caspase-9 and caspase-8, induction of cell death by Bax death pathway and Fas pathway	114
Emodin	China	Vitro	Induction of apoptosis	Hepatocellular carcinoma	Mitochondrial apoptosis pathway through cell cycle arrest and ROS generation	115
Emodin	China	Vitro	Induction of cells death	Osteosarcomama	Initiation of ROS-dependent mito-chondria-induced and ROS-independent endoplasmic reticulum stress-induced apoptosis	116
Emodin	China	Vitro	Induction of apoptosis	Lung cancer	Endoplasmic reticulum stress and the TRIB 3/NF-KB pathway	117
Emodin	China	Vitro/Vivo	Induction of apoptosis	Hepatocellular carcinoma	Mitogen-activated protein kinase (MAPK) and phosphoinositide 3-kinase (PI3K)/AKT signalling pathways	118
Emodin	China	Vitro/Vivo	Anticancer and antiproliferative	Pancreatic cancer Liver metastasis of pancreatic cancer	Inhibition of epithelial mesenchymal transition by raising increasing the content of miR-1271	119
Emodin	China	Vitro	Promotion of the arrest of cell proliferation	Lymphoma	Increase in the UHRF1D-NMT3 A-TAp73/ANp73 pathways.	120
Emodin	China	Vitro	Induction of apoptosis	Colon cancer	Induction of autophagy, during which ROS generation is of the essence.	121
Emodin	China	Vitro	Induction of growth inhibitionand apoptosis	Breast cancer	Reduction of the level of Bcl-2 and increased levels of cleaved caspase-3, PARP, p53 and Bax	122
Emodin	China	Vitro	Induction of cells growth inhibition and apoptosis	Acute myeloid leukaemia	Inhibition of the PI3K/Akt signalling pathway by activation of caspase cascades	123
Emodin	China	Vitro	Induction of apoptosis	Colon cancer	ROS is a trigger of emodin inducing apoptosis and p53 expression increases under oxidative stress, leading to Bax-mediated mitochondrial apoptosis	124
Emodin	China	Vitro	Triggers apoptosis	Neuroblastoma	Mechanism involving both reactive oxygen species and nitric oxide	125
Emodin	China	Vitro	Induction of apoptosis	Cervical cancer	Intrinsic mitochondrial and extrinsic death receptor pathways	126
Emodin	India	Vitro	Induction of apoptosis	Hepatocellular carcinoma	Blocking activation of STAT3 (Signal transducer and activator of transcription 3)	127
Emodin	China	Vitro	Induction of cells growth inhibition and apoptosis	Breast carcinoma	Modulation of the expression of apoptosis-related genes	128
Emodin	China	Vitro	Induction of apoptosis	Liver cancer	A multifaceted complex cascade of events	129
Emodin	China	Vitro/Vivo	Induction of apoptosis and inhibition of cells proliferation	Pancreatic cancer	Declining the mitochondrial membrane potential	43
Emodin	China	Vitro	Inhibition of cells proliferation and induction of apoptosis	Prostate cancer	Androgen receptor and and p53-p21 pathways and the mitochondrial pathway.	45
Emodin	China	Vitro	Inhibition of cell growth and induction of apoptosis	Leukaemia	Inhibition of phosphorylation of P210 protein, down-regulation of P210 protein expression and activation of caspase-3	130
Emodin	China	Vitro	Induction of cells growth inhibition and apoptosis	Leukaemia	Inhibition of Akt [Protein kinase B (PKB)] signal pathway	131
Emodin	Japan	Vitro	Induction of apoptosis	Multiple myeloma	Inhibition of interleukin-6-induced JAK2/STAT3 pathway	12
Emodin	Taiwan	Vitro	Induction of apoptosis	Lung adenocarcinoma	Reactive oxygen species-dependent mitochondrial signaling pathway	132
Emodin	Taiwan	Vitro	Induction of apoptosis	Promyeloleukemia	Activation of caspase 3 cascade but independent of reactive oxygen species production	133
Emodin	Indian	Vitro	Induction of apoptosis	Cervical cancer	Caspase-dependent and presumably through the mitochondrial pathway, by the activation of caspases-3, -9 and cleavage of poly (ADP-ribose) polymerase	134
Emodin	Japan	Vitro	Induction of apoptosis	Hepatocellular carcinoma	Enhancement of generation of ROS, DeltaPsim disruption and caspase activation	135
Emodin azide Methyl anthraq-uin-one derivative	China	Vitro	Inhibition of cell growth and induction of apoptosis	Breast cancer and lung adenocarcinoma with over expression of HER2/neu	Disruption of the PI3K/Akt-dependent pathway	44
Aloin	Serbia	Vitro	Antiproliferative, Cell cycle arrest in the S phase, Apoptosis	Cervical uterine carcinoma	Changes in the activity of almost all anti-oxidant enzyme	41
**Aloin**						
Aloin	China	Vitro Vivo	Inhibit of tumour angio-genesis growth	Colorectal cancer	Suppression of activation of VEGF receptor (VEGFR) 2 and STAT3 phosphorylation in endothelial cells	56
Aloin (Barbaloin)	China	Vitro/Vivo	Reduction of gastric cancer cell viability & induction of apoptosis	Gastric cancer	Induction of autophagy and ROS generation	42
Aloin	Italy	Vitro	Antineoplastic & antimetastatic	Melanoma	Induction of melanoma cell differentiation	97
**Acemannan**						
Acemannan	USA	Vivo	Anticancer	Fibrosarcoma	Macrophage activation and release of tumour necrosis factor, interl-, eukin-1 and interferon	88
Acemannan	USA	Vivo	Infiltration of tumour by immune system cells, became necrotic & regressed	Sarcoma	Stimulation of synthesis of monokines resulted in the initiation of immune attack, necrosis, and regression of tumour	91
Aloemannan	Japan	Vivo	Inhibition of tumour	Sarcoma	Not described	49
**Others**						
Dichlo-romethane	Japan	Vitro	Cells growth inhibition	Ehrlich ascites tumour	Decrease of cells in the S and G2/M phase of the cell cycle; inhibition of DNA synthesis	7
Aloesin	China	Vitro/Vivo	Induction of apoptosis, inhibition of tumour growth, migration and invasion	Ovarian cancer	Inhibition of the mitogen activated protein kinase (MAPK) signaling pathway	46
Di (2-ethylh-exyl) phthalate (DEHP)	Korea	Vitro	Growth inhibition	Leukaemia	Not described	47
Diethy-lhexyl-phthalate	Korea	Vitro	Induction of apoptosis	Leukaemia	Not described	48

Furthermore, Aloe-emodin inhibited the proliferation of Merkel Cells Carcinoma to a significant degree and has also anti-neuroectodermal tumour activity in vitro and in vivo. ^31,32^ Anthraquinones are involved in induction of death of human cancer cells in many studies.^33–36^ In Egypt, it was demonstrated that the extracts of *Aloe Vera* could have anti-hepatocarcinogenic effect through modulation of apoptosis.^37^

For hematologic cancer, it was reported that Aloe-emodin has an anti-proliferative activity in leukemia cells and in lymphoma cells.^38,39^ Moreover, it was found to have anticancer activity in multidrug resistant leukemia cells.^40^ Aloin has been reported to have an antiproliferative effect in human cervix carcinoma cells by enhancing the apoptosis^41^ and has an anti-tumour effect in gastric cancer in vitro and in vivo.^2,42^

The Emodin exerts its anti-cancer activities in pancreatic cancer cells through declining the mitochondrial membrane potential.^43^ The Emodin Azide Methyl Anthraquinone Derivative (AMAD) was found to effectively block phosphorylation of Her2/neu, suppress growth, transformation and metastasis as a tyrosine kinase inhibitor, and increase the susceptibility of Her2/neu-over expressing cancer cells to standard cytotoxic therapeutic agents. This could be a potential therapeutic strategy that may block disease pathway and improve pathology in Her2/neu-over expressing cancers.^44^ It also has an anticancer activity on prostate cancer cells.^45^

Alomicin was isolated from *Aloe arborescens* and exerted an anticancer activity in vivo for sarcoma 180 and Ehrlich ascites cancers. In mice, it inhibited 100% of sarcoma 180 at a concentration of 100mg/kg by the IP route (intraperitoneally) in DDS (Dorsal Dark Stripe) while 60% of EAC were inhibited at a concentration of 2.5mg/kg twice by the IP route. Alomicin efficiently inhibited the growth of hepatoma cells.^2^

Aloesin is an active compound of *Aloe Vera* which could arrest the cell cycle, induce apoptosis in vitro and inhibit tumour growth of ovarian cancer.^46^

The Di(2-Ethylhexyl) Phthalate (DEHP) extracted from *Aloe Vera* exerted an anti-leukaemic and anti-mutagenic effects and induced apoptosis in-vitro.^47,48^

It was reported that Aloe mannan is a polysaccha-ride extracted from *Aloe arborescens* which inhibited the growth of sarcoma implanted in mice.^49^ Administered to mouse inoculated sarcoma 180, *Aloe Vera* prolonged the life span of mouse.^50^

The mannan is extracted from Aloe Saponaria. It could inhibit tumour cell activation and proliferation and does not interfere with normal lymphocyte activation.^51^ The administration of the active compounds of *Aloe Vera* to tumour transplanted animals prolonged significantly their life. Relatively, aloe-emodin was less effective than Aloesin, Aloesin less effective than Octa-peptide and Octapeptide less effective than Barbaloin. The inhibition of cells growth depended on the compounds and the type of cancer. Indeed, the growth inhibition of Ehrlich ascites carcinoma cell number when compared to the control group followed this sequence: Aloesin < Octapeptide < Aloe-emodin < Barbaloin.^52^

#### Angiogenesis of Aloe Extracts and Compounds

Aloe-emodin was identified to have an anti-angiogenic effect.^53^ Indeed, oral administration of 150μl daily dose of *Aloe Vera* gel to mice for 3 days after L-1 sarcoma cell grafting decreased significantly the number of newly-formed blood vessels when compared with the control group.^54^ Aloe-emodin could target multiple molecules responsible for angiogenesis in colon cancer cells.^55^ Moreover, Aloin could inhibit tumour angiogenesis by blocking STAT3 activation in colorectal cancer.^56^

#### Anti-Inflammatory Activity of Aloe

It has been demonstrated that inflammation is linked to various steps involved in tumourigenesis by supplying bioactive molecules to the tumour micro-environment such as growth factors that sustain proliferative signalling, survival factors that limit cell death, proangiogenic factors, extracellular matrix-modifying enzymes that enable angiogenesis, invasion, metastasis, and inductive signals that lead to stimulation of Epithelial Mesenchymal Transition (EMT). Moreover, inflammatory cells can release substances, especially Reactive Oxygen Species (ROS), that are actively mutagenic for nearby cancer cells which accelerate their genetic evolution toward states of intensified malignancy.^5,57^ On the other hand, several studies demonstrated the anti-inflammatory activity of Aloe or its compounds in inhibiting edema in vivo.^58–61^

#### Effects of Aloe on Regulating Glucose Metabolism

Cancer cells are able to reprogram their glucose metabolism by up-regulating glucose transporters, especially GLUT1, which significantly increases glucose import into the cytoplasm. These have been demonstrated by many studies.^5^ Recently, it was proven that aloe-emodin, one of the compounds of Aloe inhibited glucose metabolism by reducing GLUT1 expression in cervical cancer Cells.^62^

#### Effects of Aloe on Cell Metastasis

Aloe-emodin could suppress the metastasis of breast cancer cells. The mechanisms is not clearly elucidated and may be related to the inhibition of invasion and migration of cells.^63^ It could also decrease protein levels of tumour metastasis-related proteins in human tongue cancer cells.^64^ *Aloe Vera* could suppress the cells proliferation in human neuroblastoma cell.^65^ Aloeemodin was involved in inhibition of key regulatory molecules in colon cancer cell migration.^55^ The antiproliferative activity of Aloe-emodin was also found in-melanoma and gastric cancer cells.^66,67^ In human hepatocellular carcinoma cells, Aloe-Emodin and its homologue emodin were able to decrease cell migration.^68^

#### Effects of Aloe on Deoxyribo Nucleic Acid (DNA Of Cancer Cells

Aloe-emodin could induced DNA damage in human lung carcinoma cells through generation of reactive oxygen species.^69^ It was also observed in leukemia cells, breast cancer cells, colon cancer cells, glioblastoma multiform cells and human embryonic kidney cells^70^. In human tongue cancer cells, this DNA damage by Aloe-emodin was followed by inhibition of DNA repair of cancer cells.^64,71^

#### Effects of Aloe on Normal Cells in Patients with Cancer

There is no cytotoxic activity towards the normal cells caused by Aloe-emodin^70^. The analysis of some studies revealed that chemotherapy is substantially better tolerated in patients concomitantly treated with Aloe.^72^ The IC_50_ of the extract of the leaf of *Aloe Vera* against breast cancer cell line was almost 15 times lower than that of *Aloe Vera* leaf extract against non-cancerous cell line.^73^ One randomised study found that oral *Aloe Vera* gel can reduce radiation-induced mucositis in head-and-neck cancer patients but did not improve tolerance to head-and-neck radiotherapy, decrease mucositis, reduce soreness, or improve patient well-being.^74^ However, it was a potential choice, for palliative treatment for patients undergoing treatment of head and neck cancer and prevent oral complications as well as oral *Aloe Vera* juice.^75,76^

On the other hand, *Aloe Vera* gel did not significantly reduce radiation-induced skin side effects. However, aqueous cream was useful in reducing dry desquamation and pain related to radiation therapy in breast cancer.^77^ In neuroectodermal tumours, *Aloe Vera* does not inhibit the proliferation of normal fibroblasts nor that of hemopoietic progenitor cells.^31^ The molecules in fluid fractions from leaf of *Aloe Vera* were found to markedly promote attachment and growth of non-neoplastic human cells, but not tumour cells. This attachment and growth of human cells is evident in natural *Aloe Vera* more than in commercial preparations may be owing to substances introduced during commercial processing.^78^

It has been reported that *Aloe vera* preparations could cause diarrhoea, hypokalemia, pseudomelanosis coli, kidney failure, phototoxicity, hypersensitive reactions and its whole leaf extracts were considered as carcinogenic in rats.^1^

#### Effects of Aloe on Telomerase Activity

Telomerase is an enzyme in control of the synthesis of telomeres and is activated in many types of cancers. In cancer cells, it promotes the replication, proliferation and metastasis of cancer cells.^79^

G-quadruplex formation might inhibit telomerase activity in most cancer cells by locking the single-stranded telomeric substrate into an inactive conformation, which is neither recognized nor elongated by telomerase. The anthraquinones were one of the first ligands found capable of stabilising G-quadruplexes and inhibiting telomerase. Aloe-emodin, Aloe-Emodin Derivative 3 (AED3) and emodin could play the same role as long as they belong to anthraquinones. Moreover, Emodin, Aloe-emodin and AED3 induced strong fluorescence quenching of 12C5TG-AgNC which indicate that they are G-quadruplex-interactive ligands.^80,81^ Furthermore, it has been reported that the Di-2-Ethylhexyl Phthalate should decrease telomerase activity and increase TNF in the rat testis.^82^ In the recent study, it has been demonstrated that Aloe-emodin is a competitive inhibitor of telomerase and a G-quadruplex structure stabiliser in breast cancer cells. It decreases the telomerase activity by competing with dNTP for binding to the enzyme active site and stabilising the telomeric G-quadruplex structure.^83^

#### Aloe and Cellular Immunity

A randomised study assessing chemotherapy alone versus chemotherapy plus *Aloe arborescens* in patients with metastatic cancer have been performed. It was reported that the lymphocyte mean number observed after therapy in patients concomitantly treated with aloe was significantly higher than that observed in the group treated with chemotherapy alone.^72^ It was also reported that aloe-emodin increased the levels of interleukin (IL)-1beta and tumour Necrosis Factor (TNF)-alpha.^84^

Aloctin A (Alo A) is an active substance of *Aloe arborescens* Miller. The treatment effects of this compound have been described in vivo and in vitro on the immune response of murine and human lymphoid cells.^85^ Alo A was also involved in inhibiting the growth of induced fibrosarcoma in mice and was not directly cytotoxic to tumour cells in vitro.^86^ Aloctin A is one of lectin plant found in Aloe. Lectin were identified to have cytotoxic effects on the tumour cell surface augments tumour-specific by enhancing immunity through activation of T cells.^87^

Acemannan is the most active polysaccharides found in *Aloe Vera*. It has been reported that this compound should exert its antitumor activity through macrophage activation and the release of tumour necrosis factor, interleukin-1, and interferon.^88,89^ The same substance Acemannan, was involved in increasing immunity in mouse whose immune systems had been damaged by radiation.^90^

Another study indicated that intraperitoneal treatment with Acemannan stimulate synthesis of monokines resulted in the initiation of immune attack (including interleukin-1 and tumour necrosis factor), necrosis, and regression of implanted sarcomas in mouse.^91^ Moreover, Acemanna (CarraVet Acemannan Immunostimulant) has been approved as a biologic treatment of fibrosarcoma in cats and dogs by the USDA.^2^

#### Aloe in combination with other cancer therapies

Aloe-emodin enhanced the activities of tamoxifen, cisplatin, doxorubicin, cyclophosphamide and 5-fluorouracil^92–94^ and Aloes Vera acts synergistically with cisplatin to inhibit proliferation of human breast and cervical cancer cells.^95^ Furthermore, some specific compounds extract from the leaf of *Aloe Vera* (such as Aloe-emodin, 7-hydroxy-2,5 dimethyl chromone, Beta-sitosterol, etc.) possess higher binding affinity toward estrogen alpha receptor than standard tamoxifen.^73^ Aloe-emodin increased the radio-sensitivity of human cervical cancer cells in vitro, inhibited their proliferation and, in combination with radiation, it induced the apoptosis.^96^ Aloin, another compound of Aloe enhanced the antineoplastic activity of cisplatin in melanoma cells^97^ and the emodin sensitised the hepatocellular carcinoma cells to the anti-tumour activity of Sorafenib (tyrosine kinase inhibitor).^98^

Aloe-emodin induced cell apoptosis and leads to cell death in vitro and in vivo while associated with photodynamic therapy it enhanced killing effect of human oral mucosa carcinoma, human gastric cancer cells and breast cancer cells.^99–101^

In their study, Lissoni P. and his colleagues compared chemotherapy alone with chemotherapy associated with Aloe. The complete response was achieved in 3% (4/121) of patients treated with chemotherapy alone versus 10% (12/119) of patients treated with chemotherapy + Aloe while partial response was achieved in 16% (19/121) of patients treated with chemotherapy alone versus 23% (28/119) of patients treated with chemotherapy + Aloe. The disease stability was observed in 31% (37/121) for patients treated with chemotherapy alone and in 34% (40/119) for patients treated with chemotherapy + Aloe. The disease progression was significantly higher in the patients treated with chemotherapy alone than in the group treated with chemotherapy + Aloe [50% (61/121) vs. 33% (39/119)].^72^

One randomised study found that oral *Aloe Vera* gel did not reduce radiation-induced mucositis in head-and-neck cancer patients.^74^ The *Aloe Vera* had no positive effect on prevalence or severity of radiation dermatitis in in breast cancer patients treated with radiation therapy.^102^ However, it was considered as an alternative agent in the treatment of mucositis induced by radiation in patients with head and neck cancers.^103^

Another study compared the administration of melatonin (hormone primarily released by the pineal gland that regulates the sleep-wake cycle) alone versus melatonin + *Aloe Vera* in patients suffering from various advanced solid tumours and for whom no effective standard anticancer therapies are available. It found a partial response achieved in 2/24 patients treated with melatonin plus Aloe and in 0/24 patients treated with melatonin alone. The disease stability was achieved in 12/24 for patients treated with melatonin plus aloe and in 7/26 for patients treated with melatonin alone. The percentage of stabilised patients was significantly higher in the group treated with melatonin + aloe than in the melatonin group (14/24 vs. 7/26). The 1-year survival patients was significantly higher in patients treated with melatonin plus aloe (9/24 vs. 4/26).^104^

It has been reported, a 64-years-old woman treated with topical *Aloe Vera* for ocular surface squamous neoplasia.^105^

In combination with surgery and radiation therapy, the Acemannan was administrated to canine (dog-like mammals) and feline (member of the cat family) suffering from fibrosarcoma and the results were impressive. While these animals had recurring disease failing previous treatment, a poor prognosis for survival, or both; the Acemannan treatment modified the tendency.^106^

*Aloe Vera* given concomitantly with honey can modulate tumour growth by reducing cell proliferation and reducing tumour weight. In fact, *Aloe Vera* may reduce tumour mass and metastasis rates, while honey may inhibit tumour growth.^107^

Emodin enhanced the antitumour effect of gemcitabine in pancreatic cancer and it could contribute to reduced chemo-resistance.^108^

## CONCLUSION

Whether the whole Aloe or its compounds are considered, we found through different articles that Aloe is a medicinal plant that has acted well against many types of cancer, namely cervix carcinoma, breast carcinoma, osteosarcoma, cancer of soft tissues, Hodgkin lymphoma, non-Hodgkin lymphoma, solid tumour of children, lung cancers, acute and chronic leukaemia, bladder cancer, ovarian cancer, gastric cancer, colon carcinoma, oral squamous cell carcinoma, colorectal cancer, lung squamous carcinoma, malignant glioma, tongue squamous cancer, prostate cancer, nasopharyngeal carcinoma, bladder cancer, hepatocellular carcinoma, Merkel cells carcinoma, leukaemia and in lymphoma, pancreatic cancer, prostate cancer, myeloma, sarcoma, hepatoma, ovarian cancer, neuroblastoma, melanoma, lung carcinoma, glioblastoma multiform, fibrosarcoma, ocular surface squamous neoplasia, pleural tumour from hepatoma, duodenal tumour, glioblastoma, hepatoma, neuroectodermal tumours, promyelocytic leukaemia, non-small cell lung carcinoma, oral mucosa carcinoma, pancreatic cancer, liver metastasis of pancreatic cancer, lymphoma, acute myeloid leukaemia, liver cancer, multiple myeloma, lung adenocarcinoma, promyeloleukeamia, breast cancer and lung adenocarcinoma with overexpression of HER2/neu, Ehrlich ascites tumour and fibrosarcoma.

Moreover, this review points out the fact that Aloe or at least one of its compounds could interrupt the pro-growth signalling pathways of cancer and this is the first time to show the therapeutic effect of Aloe on cancer based on biological capabilities of cancer. This can lead to development of a drug based on the whole Aloe or their compounds

In fact, a part from whole Aloe, 9 different compounds of different Aloe have been identified to have anticancer activities involving many pathways. Anticancer activity depended variably on compounds types, time and type of cancer. Given the anticancer effects of its compounds taken separately or the whole leaf, Aloe exerts the anticancer effects through many mechanisms which could act synergistically. There is a high potential that-whole Aloe or the combination of some of its compounds could be a chemotherapy based treatment which could have a therapeutic value in chemotherapy of different types of cancers with no or minimum side effects.

However, we realised that there are few studies conducted on Aloe illustrating its molecular suppressive activity of cancer. It is for instance the role of Aloe on inflammation, regulating glucose metabolism in cancer cells. Only 2 databases were explored and moreover many of these studies were conducted on *Aloe vera* despite the fact that there are many species of Aloe and this is one of the weak points of this review. There is the need for more studies especially in vivo to be undertaken to examine the molecular activities of the different species of Aloe so that more effective therapeutics could be designed.
